# Artificial intelligence (AI) in personalized medicine: AI-generated personalized therapy regimens based on genetic and medical history: short communication

**DOI:** 10.1097/MS9.0000000000001320

**Published:** 2023-09-13

**Authors:** Ad-Duhaa E. Parekh, Omer A. Shaikh, Simran, Sadia Manan, Md. Al Hasibuzzaman

**Affiliations:** a Department of Medicine, Jinnah Sindh Medical University; b Department of Medicine, Ziauddin University, Karachi; c Department of Medicine, Shaheed Mohtarma Benazir Bhutto Medical University, Larkana, Pakistan; d Ningbo First Hospital, Ningbo, Zhejiang, China; e Institute of Nutrition and Food Science, University of Dhaka, Dhaka, Bangladesh

**Keywords:** AI, genetic, personalized medicine

## Abstract

Personalized medicine entails tailoring medical care to an individual's unique genomic and molecular characteristics. AI holds significant promise in advancing the field of personalized medicine. The challenge lies in effectively analyzing vast amounts of data to create tailored treatment approaches. The incorporation of AI into personalized treatment will require healthcare infrastructure adjustments. Upon patients' arrival, their personal data and clinical information (including images, electrophysiology findings, genetic data, blood pressure, medical notes, etc.) are gathered into the AI system with their consent. Subsequently, the AI system utilizes this patient-specific data to offer healthcare recommendations, aiding healthcare professionals in their clinical decision-making. Results and insights from these recommendations, whether accurate or not, are logged and fed back into the AI system to enhance its precision.

The concept of personalized medicine – that medical care can be customized to an individual’s genomic and molecular profile – has far-reaching implications that go beyond the technology that makes it feasible. It will necessitate changes in healthcare infrastructure, diagnostics and medicines business models, payment policy from government and private payers, and a new regulatory supervision strategy. Personalized medicine will also transform medical practices from reactive disease treatment to proactive healthcare management, including screening, early treatment, and prevention, and will change the responsibilities of both the physician and the patient. It would increase reliance on electronic medical records and decision support tools in an industry that has a long history of anti-information-technology hostility^1^. Given the importance of data-intensive assays in revealing appropriate intervention targets and strategies for personalizing medicines, artificial intelligence (AI) can play a critical role in the development of personalized medicines at all relevant stages of clinical development and implementation of new personalized health products, from identifying appropriate intervention targets to testing their utility^2^.

Personalized medicine employs particular medical information to develop strategies and therapies for recognizing and treating illnesses in order to maintain people’s health^3^. In this type of medical practice, doctors combine the results of all patient data that is at their disposal such as symptoms, the results of conventional tests, the patient’s medical and family history, and some genomic data in order to make accurate diagnoses and develop tailored treatment plans in accordance with those findings^1^. Professionals in health information management (HIM), who are in charge of handling patient data, will be essential to this individualized healthcare model justifying the importance of AI. The authors of a recent National Academy of Medicine report on the current and future state of AI in healthcare noted ‘unprecedented opportunities’ to augment the care of specialists, as well as the assistance that AI provides in combating the realities of being human (such as fatigue and inattention) and the risks of machine error. Importantly, the article emphasizes that, while these technologies must be used with caution, they hold great promise. But the problem that AI in personalized medicine aims to solve is the challenge of analyzing large amounts of data to develop personalized treatment plans. Traditional personalized medicine approaches have limitations due to the complexity of analyzing the vast amounts of data involved in creating a personalized treatment plan. However, utilizing AI to find patterns and correlations in data can assist overcome these constraints, which can then be utilized to generate more accurate and successful personalized treatment regimens^4^.

Doctors have requested whole-genome sequencing for their patients in an attempt to uncover genetic explanations for some ailments that cannot be diagnosed using conventional methods, and other healthcare experts have begun to analyze genomic data^5^. In December 2013, the US Food and Drug Administration (FDA) approved the first high-throughput (next-generation) genomic sequencer, Illumina’s MiSeqDx. This marketing authorization is an important milestone in the process of exploiting genetic data in a healthcare setting since it allows for the development and use of several novel genome-based tests. The FDA and the National Institute of Standards and Technology collaborated to create the whole human genome DNA and the best sequence interpretation of such genomes in order to generate genomic reference materials for performance evaluation^6^. They may also arrange genetic tests at the request of patients if there is supporting evidence for the patient’s case. Physicians rely on HIM experts to gather knowledge regarding existing genetic tests, as well as their limitations and implications. This assists both patients and professionals in determining the best current treatment alternatives.

HIM experts must also keep patient data up to date because new investigations may disclose new facts. They must be trained to do this task. Powerful information systems are specialized to the administration of large-scale biological data. For example, openBIS (open-source Biology Information System) is a distributed information system that may be used to manage DNA sequences obtained by next-generation sequencing methods^7^.

Image analysis, medicine discovery, and diagnostics are just a few of the applications of AI in healthcare. Data-intensive biomedical technology in study has also shown that people vary substantially in terms of disease processes and response to treatment on genetic, biochemical, physiological, exposure, and behavioral levels. This suggests that it is necessary to adjust, or ‘personalize,’ medications so that they work better for the specific needs of each patient. Through a symbiotic relationship with the use of data-intensive assays, AI can play a crucial role in developing personalized medicine and discovering relevant intervention targets to evaluate their effectiveness^2^. The discovery of blood types and its effect on blood transfusions in the early twentieth century is considered the earliest example of personalized medicine. Personalized medicine improved transfusion safety by matching blood donors and recipients^8^.

However, the requirement for vast amounts of high-quality data, potential bias in data analysis, and ethical concerns about privacy and security are among the problems and limitations of employing AI in personalized medicine. Personal genetic data analysis for healthcare reasons is complex due to the availability of large genomic datasets and the requirement for highly qualified genomic data analysts, databases, algorithms, software programmes, and computer resources. Most clinicians do not have access to these resources. Even if one can easily access excellent genomic data analysis programs and powerful workstations, it is still challenging for most healthcare professionals to select the best program and the correct parameters for that particular data set because they typically do not have the required training in this field.

When attempting to employ personal genomic information in customized medicine, the accuracy of the data obtained from raw datasets is the most pressing challenge. To detect and reduce bias in data and models, IBM has developed an online toolbox (AI Fairness 360) to assist researchers in examining bias among datasets and models, as well as methods to mitigate bias in classifiers. Model performance and therapeutic efficacy may be influenced by socio-environmental factors and workflows where the AI model will be implemented. Data security and privacy are critical for verifying AI models in the clinical setting and considering an iteration loop before generally adopting them. Promising results were reported by Baowaly and colleagues, but more work needs to be done in AI to guarantee data privacy and security. Other challenges include high-throughput technologies that generate a large amount of genomic data, which can be difficult to manage and analyze. Additionally, the quality of the data can be poor, which can lead to inaccurate results. Personal genomic data are sensitive information that needs to be protected. There are policies and laws related to the management of personal genomic information. Technical applications such as Interpretome and GenePING aim to protect personal genomic information. There are also legal, social, and ethical issues related to personal genomic information. The Genetic Information Nondiscrimination Act (GINA) is the law specifically created to protect individuals from discrimination based on their genetic test results. GINA extended the medical privacy and confidentiality rules to the disclosure of genetic information before the modifications of HIPAA (Health Insurance Portability and Accountability Act) and the HITECH (Health Information Technology for Economic and Clinical Health Act). GINA represents a landmark in the field of personal genomics because it removes one important concern when patients consider taking genetic tests. It is also beneficial for biomedical research because it removes similar concerns for participants of genetic research. Physicians rely on HIM professionals to collect information about available genetic tests and their limitations or consequences creating issues relevant to HIM professionals. These professionals are also responsible for updating patient data regularly because new investigations may reveal new information. They need to be trained to perform this job^7^.

To overcome these challenges, potential solutions include increasing collaboration and data sharing among healthcare providers, implementing rigorous quality control measures, and developing ethical guidelines for AI in healthcare. Scientists now have a greater knowledge of the association between genetic changes in patients’ genomes and their risk of getting specific diseases, as well as their possible responses to various treatment regimens, thanks to considerable advances in research. On the other side, these thorough study findings also make it difficult to assess individual genomic data for medical needs. Given that genomics research will very certainly one day allow more information to be extracted from a human genome, the threat to the individual’s progeny may be even more serious. Hence, before the widespread use of genomic data in clinical practice, a more robust and sophisticated security framework should be put in place to protect personal genomic data. It is the duty of all healthcare professionals to inform the public about the advantages of customized therapy and the risks associated with genetic testing. To figure out how to incorporate genetic data into medical record systems for clinical decision support, software engineers must collaborate closely with healthcare providers. To assist doctors in implementing genomics into their practices, it could be advantageous to create a national information infrastructure with specified standards in place. Physicians must increase their understanding of genomics in order to properly order genetic tests and genomic analyses, comprehend the outcomes of such tests and analyses, and customize the course of treatment for each patient^7^.

One suggestion for managing the overwhelming volume of genomic data is to apply a compression algorithm to reduce the sizes of these sequences. This can significantly reduce the size of stored data. Another approach is to keep only one reference genome and record all the differences between other human genomes and this reference genome. This approach can also significantly reduce the size of stored data. Some information systems are specifically dedicated to the management of large-scale biological information. For instance, openBIS is a distributed information system that can be used for managing DNA sequences generated by next-generation sequencing technologies. One essential component of HIM training is to educate HIM students on how to manage, protect, and apply genomic data in clinical settings. HIM professionals may also need to build knowledge-based decision-making systems for genomics-based personalized medicine practices so that clinicians can extract the most needed information from complex genomic data. The Office of the National Coordinator for Health Information Technology (ONC) is currently working on integrating genomic information into health information technology systems in order to take advantage of the full potential of genomic information in healthcare^7^.

In conclusion, the future of AI in personalized medicine is promising, with the potential to revolutionize the way healthcare is delivered. Future research should prioritize the development of more accurate and efficient AI algorithms, the improvement of data quality and access, and the resolution of ethical and privacy concerns. AI has the ability to greatly enhance patient outcomes and overall healthcare quality if these problems and constraints are overcome. In this discipline, the IBM Watson system is a pioneer. The system, which contains both ML (Machine Learning) and NLP (Natural Language Processing) modules, has shown encouraging results in oncology. In a cancer study, for example, 99% of Watson’s therapy suggestions agree with medical conclusions. In addition, Watson worked with Quest Diagnostics to provide the AI Genetic Diagnostic Analysis. Furthermore, the system began to have an impact on actual clinical practices. Watson, for example, successfully detected a rare secondary leukemia caused by myelodysplastic syndromes in Japan by studying genetic data. One prototype for connecting an AI system with front-end data input and back-end clinical actions is the cloud-based CC-Cruiser. More specifically, when patients arrive, their demographic information and clinical data (pictures, EP results, genetic results, blood pressure, medical notes, and so on) are collected into the AI system with their permission. The AI algorithm then uses the patients’ data to provide healthcare recommendations. These recommendations are delivered to clinicians to help them make clinical decisions. Feedback on the ideas (whether correct or incorrect) will also be recorded and put back into the AI system so that it can continue to improve accuracy^9^.

## Ethical approval

Ethics approval was not required for this commentary article.

## Consent

Informed consent was not required for this commentary article.

## Sources of funding

The author(s) received no financial support for the research, authorship, and/or publication of this article.

## Author contribution

A.-D.E.P., O.A.S., F.S., S.M. and M.A.H.: literature search and manuscript preparation. M.A.H.: conceptualization, methodology, and supervision. All authors read and approved the final manuscript.

## Conflicts of interest disclosure

The author(s) declared no potential conflicts of interest concerning the research, authorship, and/or publication of this article.

## Research registration unique identifying number (UIN)

Not applicable.

## Guarantor

Md. Al Hasibuzzaman.

## Data availability statement

Not applicable.

## Provenance and peer review

Not commissioned, externally peer-reviewed.
